# Protocol for evaluating the activity of R2 retrotransposons in mammalian cells

**DOI:** 10.1016/j.xpro.2024.103538

**Published:** 2025-01-06

**Authors:** Yangcan Chen, Shengqiu Luo, Yanping Hu, Qi Zhou, Wei Li

**Affiliations:** 1 Key Laboratory of Organ Regeneration and Reconstruction, State Key Laboratory of Stem Cell and Reproductive Biology, Institute of Zoology, Chinese Academy of Sciences, Beijing 100101, China; 2 Institute for Stem Cell and Regeneration, Chinese Academy of Sciences, Beijing 100101, China; 3 University of Chinese Academy of Sciences, Beijing 100049, China; 4 Bejing Institute for Stem Cell and Regenerative Medicine, Beijing 100101, China

**Keywords:** molecular biology, CRISPR, biotechnology and bioengineering

## Abstract

R2 retrotransposons can be harnessed to insert genes at targeted sites by all-RNA delivery, presenting a new technology for next-generation biotherapeutics. Here, we report a protocol for evaluating the gene integration activity of R2 retrotransposons in mammalian cells. We describe the construction of vectors separately expressing R2 protein and donor, the process of liposome transfection, and flow cytometry. This protocol provides a useful reporter system, which can be applied to evaluate the activity of other new retrotransposon systems.

For complete details on the use and execution of this protocol, please refer to Chen et al.^1^

## Highlights

• A GFP-intron reporter system for screening R2 elements in mammalian cells
• Construction of vectors separately expressing R2 protein and donor RNA
• Quantification of integration efficiency of R2 systems via flow cytometry

## Abstract (editor highlights)

Publisher’s note: Undertaking any experimental protocol requires adherence to local institutional guidelines for laboratory safety and ethics.

## Graphical abstract

**Figure:**
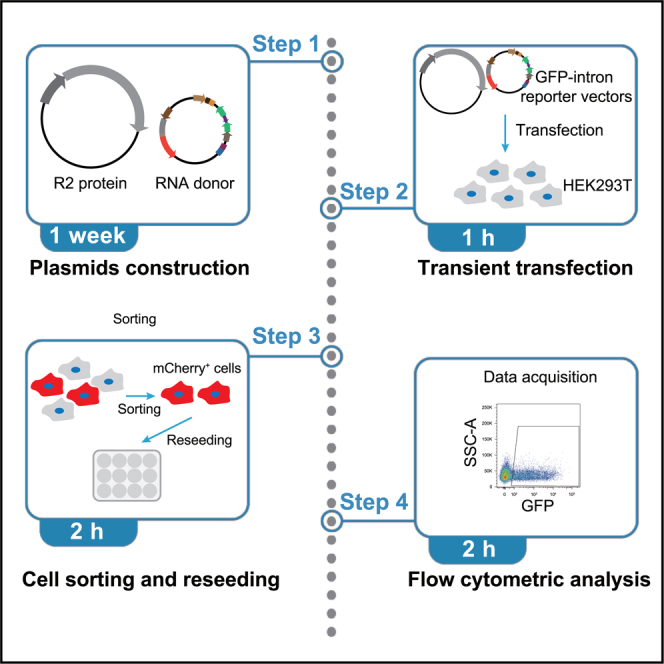

## Before you begin

**Figure 1:**
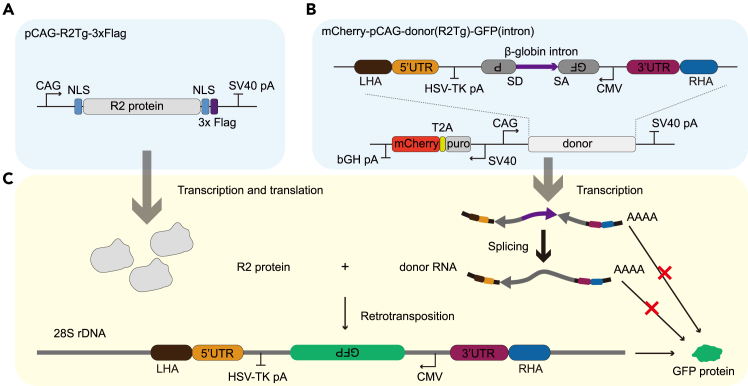
GFP-intron reporter system used for accessing the activity of R2 retrotransposition (A) Construction of plasmid expressing the R2 protein. CAG, a cytomegalovirus major immediate-early enhancer combined with the chicken beta-actin promoter; NLS, nuclear localization signals; SV40 pA, Simian virus 40 polyadenylation signal. The promoter or pA signal above the line represents the plus strand. (B) Construction of plasmid expressing RNA donor. LHA, left homologous arm; HSV-TK pA, Herpes simplex virus type 1 thymidine kinase polyadenylation signal; SD, splicing donor; SA, splicing acceptor; CMV, cytomegalovirus immediate early promoter; RHA, right homologous arm; bGH pA, bovine growth hormone polyadenylation signal; SV40, Simian virus 40 promoter. The promoter or pA signal above the line represents the direction of the plus strand while below the line represents the direction of the minus strand. (C) Schematic diagram of evaluating the activity of R2 element for inserting full-length GFP gene into mammalian cells.

R2 retrotransposons rely on reverse transcription of RNA intermediates to propagate and site-specifically integrate into the 28S rDNA loci in their natural host. These characteristics make it possible for R2 retrotransposons to insert large-fragment genes at targeted sites by all-RNA delivery, presenting a new technology for next-generation biotherapeutics. Here, we provide a GFP-intron reporter system to evaluate the activity of the R2 retrotransposons in mammalian cells. Unlike classical all-in-one retrotransposon reporter construct,^2^^,^^3^ this system includes two plasmids, one plasmid for expressing the R2 protein and the other for expressing the R2 donor RNA (Figures 1A and 1B). For the RNA donor plasmid, we designed a GFP-intron cassette, in which the GFP gene is separated by an invertedly-inserted β-globin intron. Inverted intron prevents GFP expression from one promoter, while antisense direction of ORF prevents its expression from the other. Only when the R2 system reverse transcribes the intron-less GFP anti-sense mRNA into DNA and integrates it into the genome, can the GFP protein be expressed (Figure 1C). We placed an SV40-puro-T2A-mCherry cassette on the plasmid expressing RNA donor, so we can use fluorescence activated cell sorting (FACS) to enrich successfully-transfected cells. For the plasmid expressing R2 protein, we added NLS sequences at both the N-terminus and the C-terminus of the protein to facilitate its nuclear entry.

In this protocol, we focused on R2Tg retrotransposon, which is from the genome of *Taeniopygia guttata*, as an example to implement the experiments.^1^

***Note:*** Replace the sequences of R2 protein coding sequence, 5′ UTR and 3′ UTR when using this reporter system to screen different R2 elements.

## Key resources table

**Table:** 

REAGENT or RESOURCE	SOURCE	IDENTIFIER
**Bacterial and virus strains**		

Fast-T1 competent cell	Vazyme	Cat#C505-03

**Chemicals, peptides, and recombinant proteins**		

DMEM	Gibco	Cat#C11995500BT
FBS	Gibco	Cat#10099141
Penicillin-Streptomycin (Pen/Strep)	Gibco	Cat#15140122
Trypsin-EDTA (0.25%), phenol red	Gibco	Cat#25200072
Opti-MEM I reduced serum medium	Gibco	Cat#31985088
Phosphate-buffered saline (1×)	Pricella	Cat#PB180327
Lipofectamine 3000 Transfection Reagent	Invitrogen	Cat#L3000015
NEBuilder HiFi DNA Assembly Master Mix	NEB	Cat#E2621L
Q5 Hot Start High-Fidelity 2× Master Mix	NEB	Cat#M0494S
EcoRI-HF	NEB	Cat#R3101L
XmaI	NEB	Cat#R0180L
BamHI-HF	NEB	Cat#R3136L
NheI-HF	NEB	Cat#R3131L
Poly-D-lysine	Sigma	Cat#P6407
rCutSmart Buffer	NEB	Cat#B6004S
Tryptone	Oxoid	Cat#LP0042
NaCI	Sinopharm Group	Cat#10019318
Yeast extract	Oxoid	Cat#LP0021
Agar	Dingguo Changsheng Company	Cat#DH010-1.1
Ampicillin sodium salt	Amresco	Cat#0339-25G

**Critical commercial assays**		

TIANpure Midi Plasmid Kit	Tiangen	Cat#DP107–02
Zymoclean Gel DNA Recovery Kit	Zymo Research	Cat#D4008

**Experimental models: Cell lines**		

HEK293T	Pricella	Cat#CL-0005

**Recombinant DNA**		

pCAG-R2Tg-3xFlag	Chen et al.^1^	#223250
pCAG-R2Tg^opt^-3xFlag	Chen et al.^1^	#223251
mCherry-pCAG-donor(R2Tg)-GFP(intron)	Chen et al.^1^	#223248
mCherry-pCAG-donor^opt^(R2Tg)-GFP(intron)	Chen et al.^1^	#223249
mCherry-pCAG-mock	Chen et al.^1^	#228504
pCAG-protein-mock	Chen et al.^1^	#228505

**Software and algorithms**		

BD FACSDiva	BD Biosciences	https://www.bdbiosciences.com/
FlowJo	BD Biosciences	https://www.bdbiosciences.com/
Leica application suite X	Leica	https://www.leica-microsystems.com/

**Other**		

24-well plates	Corning	Cat#3524
12-well plates	Corning	Cat#3513
5 mL round bottom polystyrene test tube	Falcon	Cat#352003
Countess II FL automated cell counter	Invitrogen	Cat#AMQAF1000
Leica DMi8 microscope	Leica	N/A
BD FACSAria Fusion instrument	Beckman Coulter	N/A
BD LSRFortessa instrument	Beckman Coulter	N/A

## Materials and equipment

**Table:** HEK293T cell culture medium

Reagent	Final concentration	Amount
DMEM	89% (v/v)	445 mL
FBS	10% (v/v)	50 mL
Penicillin-Streptomycin (Pen/Strep)	1% (v/v)	5 mL
**Total**	**N/A**	**500 mL**

Store HEK293T cell culture medium at 4°C for up to 4 weeks.

**Table:** Luria-Bertani (LB) medium

Reagent	Amount
Tryptone	10 g
NaCI	10 g
Yeast extract	5 g
ddH_2_O	Up to 1 L

Autoclave the LB medium and store it at 4°C for up to 4 weeks. Prepare 1 L of LB solid medium by adding 15 g of agar to the recipe. Prepare ampicillin selective medium by adding ampicillin antibiotic (the final concentration is 100 μg/mL) to the medium. Store solid plates at 4°C for up to 2 weeks.

## Step-by-step method details

### Construction of GFP-intron reporter vectors

**Timing: 1 week**

This step describes the process of constructing the GFP-intron reporter vectors using NEBuilder HiFi DNA Assembly. The reporter vectors include pCAG-R2Tg-3xFlag (Addgene #223250) and mCherry-pCAG-donor(R2Tg)-GFP (intron) (Addgene #223248) plasmids (Figure 1).

1. Obtain all fragments for vectors construction by PCR.
  a. Synthesize human codon-optimized DNA sequences (optimized by GenScript) for R2 protein and DNA sequences of GFP, UTRs and the β-globin intron. The synthetic sequences are listed in Table S1.
    ***Note:*** The 5′ UTR, 3′ UTR and R2 protein sequences of R2 elements can be obtained from the Repbase database.^4^
  b. PCR amplification for synthesized DNA fragments. The primer sequences are listed in Table S1.
    **Table:** PCR reaction master mix

Reagent	Final concentration	Amount
DNA template	1.25–2.5 ng/μL	1 μL (50–100 ng/μL)
Q5 Hot Start High-Fidelity 2× Master Mix (NEB)	1×	20 μL
Forward Primer	0.5 μM	1 μL (20 μM)
Reverse Primer	0.5 μM	1 μL (20 μM)
ddH_2_O	N/A	17 μL
    **Table:** PCR cycling conditions

Steps	Temperature	Time	Cycles
Initial Denaturation	95°C	5 min	1
Denaturation	95°C	20 s	35 cycles
Annealing	Depend on primer pair	20 s	
Extension	72°C	10 s / 1 kb	
Final extension	72°C	5 min	1
Hold	4°C	–

    ***Note:*** For different fragments, the extension time is dependent on the size of DNA fragment, usually 10 s per kb.
  c. Separate PCR products by 1.5% agarose electrophoresis.
  d. Recover fragments using a Zymoclean Gel DNA Recovery Kit (Zymo Research). Follow the steps of recommended protocol.
2. Prepare backbones.
  a. Digest pCAG-3×Flag backbone with XmaI and BamHI-HF. Digest mCherry-pCAG-SV40pA backbone with EcoRI-HF and NheI-HF. The sequences of backbone plasmids are listed in Table S1 (Addgene #228504, #228505).
    **Table:** Restriction endonuclease reaction master mix

Reagent	Final concentration	Amount
Endonuclease 1 (e.g.,: EcoRI-HF)	1000 units/mL	5 μL (20,000 units/mL)
Endonuclease 2 (e.g.,: NheI-HF)	1000 units/mL	5 μL (20,000 units/mL)
rCutSmart Buffer (10 ×)	1 ×	10 μL
Template plasmid	50 μg/mL	5 μL (1 μg/μL)
ddH_2_O	N/A	75 μL
**Total**	**N/A**	**100 μL**
    **Table:** Reaction conditions

Steps	Temperature	Time
Incubation	37°C	3 h
Hold	4°C	–
  b. Separate cleaved backbones by 1.5% agarose electrophoresis.
  c. Recover backbone fragments using a DNA gel extraction kit such as Zymoclean Gel DNA Recovery Kit (Zymo Research). Follow the steps of recommended protocol.
3. To construct R2 protein expression plasmid, join DNA fragment of R2 protein ORF in a pCAG-3×Flag backbone by Gibson assembly. To construct RNA donor expression plasmid, join PCR-amplified fragments related to R2 donor in a mCherry-pCAG-SV40pA backbone by Gibson assembly.
  a. Prepare DNA Assembly reaction master mix.
    **Table:** NEBuilder HiFi DNA Assembly reaction master mix

Reagent	Amount
Total PCR fragments	N× 1 μL (50–200 ng/μL)
Backbone	1 μL (50–200 ng/μL)
NEBuilder HiFi DNA Assembly master mix (NEB)	N+1 μL

    ***Note:*** "N" represents the number of total PCR fragments. The recommended DNA molar ratio is usually 1:1 to 1:5 for backbone to each fragment.
    **Table:** Reaction conditions

Steps	Temperature	Time
Incubation	50°C	20 min∗
Hold	4°C	–

∗Note: Increase the incubation time when assembling more fragments. Usually when *N* ≤ 2, incubating for 20 min. When *N* > 2, for each additional fragment, the incubation time is increased by 10 min.
  b. Add all reaction volume to 50 μL Chemically competent cells (for example, Fast-T1 competent cells from Vazyme). Pipet the mixture up and down gently. Do not vortex.
  c. Place the mixture on ice for 20 min.
  d. Heat shock at 42°C for 60 s.
  e. Place tubes on ice for 2 min.
  f. Pipet the mixture onto the selection plates (Luria-Bertani solid medium, ampicillin resistance) and apply evenly using a coating stick.
  g. Incubate the plates 12–16 h at 37°C.
4. Select clones for Sanger sequencing (commissioned to Tianyi Huiyuan Biotechnology Co., Ltd.), and choose the correct clone.
5. Add the correct clone to 15 mL LB medium with 100 μg/mL ampicillin and place samples in a shaker for 12–16 h at 37°C and 220 rpm.
6. Extract plasmids using TIANpure Midi Plasmid Kit (Tiangen) following the recommended protocol after Sanger sequencing verification.

***Note:*** Plasmids can be stored at −20°C for long term storage (over 6 months).

### Transient transfection

**Timing: 1 h**

This step details the process of transient transfection of reporter vectors into HEK293T cells.

7. Plate coating.
  a. Add 250 μL poly-D-lysine (PDL) at a concentration of 0.1 mg/mL to 24-well plates for 15 min before seeding cells.
  b. Remove the PDL solution.
  c. Wash twice with 250 μL 1× phosphate-buffered saline (PBS).
    ***Note:*** Make sure to completely cover each well with 250 μL 1× PBS because of the cytotoxicity of PDL.
8. Cell preparation before transfection.
  a. Culture HEK293T cells using HEK293T cell culture medium in a 37°C incubator with 5% CO2. Seed approximately 4 × 10^6^ cells in a 10 cm dish and passage every two days.
  b. Wash HEK293T cells with 3 mL 1× PBS once.
  c. Add 3 mL 0.25% Trypsin-EDTA (Gibco) and incubate at 37°C for 3 min.
  d. Add equal HEK293T cell culture medium (3 mL) and transfer the total cell suspension to 15 mL centrifuge tube.
  e. Centrifuge the cells at 300 × *g* for 3 min.
  f. Remove supernatant, then resuspend cells with 1 mL HEK293T cell culture medium.
  g. Count total live cells by a cell counter.
    i. Prepare the sample by mixing 10 μL of cell suspension and 10 μL of 0.4% trypan blue stain.
    ii. Pipet 10 μL the trypan blue-stained sample into a Countess chamber slide.
    iii. Count live cells by a cell counter, e.g., Countess II FL automated cell counter.
  h. Seed approximately 3 × 10^5^ live HEK293T cells in a 24-well PDL coated plate. Make sure cells are about 70% confluence when transfection.^5^^,^^6^
9. Transfection. For experiments of screening new R2 systems, transfect 250 ng pCAG-R2X-3xFlag plasmid and 250 ng mCherry-pCAG-donor(R2X)-GFP(intron) plasmid to HEK293T cells via Lipofectamine 3000 (Invitrogen) (The R2X means new R2 system, such as R2Tg). The brief steps are as follows.
  a. Dilute 1.5 μL Lipofectamine 3000 Reagent in 25 μL Opti-MEM Medium. Pipet the mixture up and down gently.
  b. Prepare mixture of DNA by adding 250 ng pCAG-R2X-3xFlag plasmid and 250 ng mCherry-pCAG-donor(R2X)-GFP(intron) plasmid to 25 μL Opti-MEM Medium, then add 1 μL P3000 Reagent. Pipet the mixture up and down gently.
  c. Add all of the diluted DNA to the tube of diluted Lipofectamine 3000 Reagent. Pipet the mixture up and down gently and incubate for 15 min at room temperature (about 20°C).
  d. Pipette dropwise the DNA-lipid complex evenly to cells and shake the wells for even distribution.

### Cell sorting and reseeding

**Timing: 2 h**

This step details the process of sorting mCherry^+^ cells and seeding in 12-well plates.

10. Prepare cells 1 day after transfection.
  a. Remove supernatant and wash the cells with 250 μL 1× PBS.
  b. Detach the cells with 200 μL pre-heated (37°C) 0.25% Trypsin-EDTA at 37°C for 3 min.
  c. Add equal HEK293T cell culture medium (200 μL), collect all liquid to a 1.5 mL tube and centrifuge the cells at 300 × *g* for 3 min.
  d. Remove supernatant, then resuspend cells with 120 μL HEK293T cell culture medium and transfer cells to a 5 mL round bottom tube.
11. Sorting mCherry^+^ cells.
  a. Set up flow cytometer. Use the untreated samples (without transfection) to set up the parameter. Set up gating strategy to collect mCherry^+^ cells. Keep the cell samples on ice while setting up the sorter.
    ***Note:*** Establish gates using untreated control cells. Use forward scatter and side scatter plots (SSC-A × FSC-A) to exclude debris and gate cell populations. Use side scatter width and side scatter area plots (SSC-W × SSC-A) to exclude cell doublets. Draw gates to collect subsets of mCherry^+^ cells based on the positive threshold established using untreated cells (SSC-A × PE-CF594-A). Subsequently, sort the cells with the top 40% of mCherry^+^ signal.
  b. Sort approximately 50,000 mCherry^+^ cells using BD FACSDiva software by a BD FACSAria Fusion instrument (Beckman Coulter).
    ***Note:*** Keep aseptic operations when sorting cells.
12. Reseed the collected cells.
  a. Transfer the collected cells into 1.5 mL tubes and centrifuge the cells at 300 × *g* for 3 min at room temperature (about 20°C).
  b. Remove supernatant, then resuspend cells with 500 μL HEK293T cell culture medium. Add the cells and another 1.5 mL cell culture medium into 12-well plates.
  c. Culture cells for additional 6 days, without cell passaging or media changes.

### Flow cytometric analysis

**Timing: 2 h**

This step describes how to analyze the results of cell samples. Seven days after transfection and before treatment of cells, the plates can be placed under a fluorescence microscope to observe the expression of GFP.

13. Detach and collect HEK293T cells seven days after transfection for flow analysis.
  a. Remove supernatant and wash the cells with 250 μL 1× PBS.
  b. Detach the cells with 200 μL pre-heated 0.25% Trypsin-EDTA at 37°C for 3 min.
  c. Add equal HEK293T cell culture medium (200 μL), collect all liquid to a 1.5 mL tube and centrifuge the cells at 300 × *g* for 3 min.
  d. Remove supernatant, then resuspend cells with 300 μL 1× PBS and transfer cells to a 5 mL round bottom tube.

***Note:*** If there is any need for subsequent experiments using these samples, such as genotyping, samples can be harvested at Step 13d.

14. Data acquisition.
  a. Set up flow cytometer. Use negative control cells (transfected donor-expressing plasmid only) to set up the parameter. Set up gating strategy to collect GFP^+^ cells.
    ***Note:*** Establish gates using negative control cells (transfected donor-expressing plasmid only). Use forward scatter and side scatter plots (SSC-A × FSC-A) to exclude debris and gate cell populations. Use forward scatter height and forward scatter area plots (FSC-H × FSC-A) to exclude cell doublets. Draw gates to collect subsets of GFP^+^ cells based on the positive threshold established using negative control cells (SSC-A × FITC-A).
  b. Analyze approximately total 30,000 cells to acquire data by a BD LSRFortessa instrument (Beckman Coulter).
15. Use the FlowJo v.10 software to analyze the results.

## Expected outcomes

**Figure 2:**
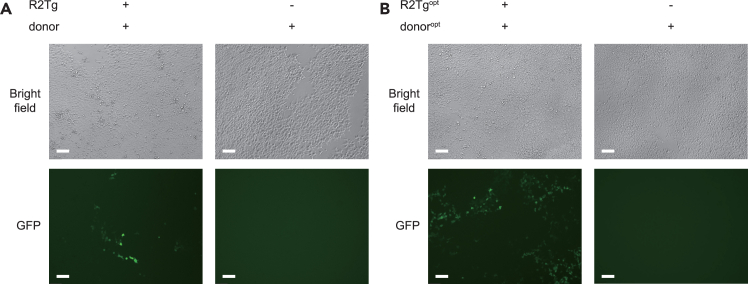
Fluorescent images showing the GFP expression in HEK293T cells treated by R2 systems Images of HEK293T cells treated by R2 systems with the observable full-length GFP gene insertion activity, including wt-R2Tg (A) and en-R2Tg (B) system. Scale bars, 100 μm.

**Figure 3:**
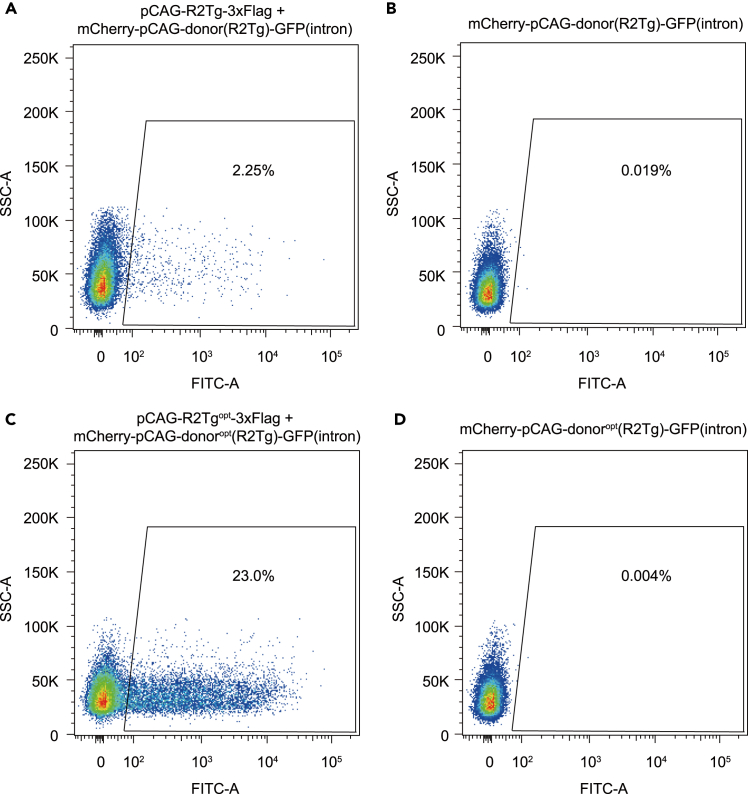
Flow cytometry showing the gene integration efficiency of wt-R2Tg and en-R2Tg systems (A and B) Efficiency of wt-R2Tg system (A) and its related control sample (B). SSC, side scatter; FITC, Fluorescein isothiocyanate. A means area. (C and D) Efficiency of en-R2Tg system (C) and its related control sample (D).

Following this protocol, we can discover the R2 systems that are active in mammalian cells. Using a Leica DMi8 microscope, we can observe GFP expression in cells treated with wt-R2Tg and en-R2Tg systems^1^ (Figures 2A and 2B). The wt-R2Tg system contains plasmids expressing wild-type R2Tg and its donor, which are constructed in this protocol. The en-R2Tg system contains plasmids expressing optimized R2Tg (R2Tg^opt^) and donor (R2Tg^opt^), which are generated by our previous work.^1^ After acquiring data using flow cytometry, we can quantify the efficiency of gene integration according to the proportion of fluorescence-positive cells. Figure 3 shows an example of testing the activity of wt-R2Tg and en-R2Tg systems. The efficiency of wt-R2Tg and en-R2Tg systems were respectively 2.25% and 23.0% (Figures 3A and 3C), when the samples transfected only with donor plasmid were used as controls (Figures 3B and 3D).

## Limitations

This protocol offers an easy-to-implement reporter system for assessing the gene integration efficiency of R2 elements in mammalian cells. The proportion of GFP-positive cells can accurately reflect the full-gene integration activity of retrotransposons. However, it should be noted that, in this reporter system, flow cytometry analysis of GFP fluorescence alone is unable to display the portion of the partially integrated GFP gene that is truncated and incapable of expressing GFP proteins, unless genotyping or DNA sequencing methods are used.^1^^,^^3^ The truncations of 5′ end, which are common in R2 elements, generally represent one of the most diagnostic features of non-LTR retrotransposons.^7^ Moreover, the exact copy number of the integrated transgene cannot be quantified by this protocol and should be detected by other methods such as digital quantitative PCR.^1^^,^^8^ In this study, “no R2 protein” conditions were used as controls. To rule out non-retrotransposition-mediated GFP expression, it can be considered to use “R2 protein variants” with inactivated reverse transcriptase domain or endonuclease domain for stricter controls.

## Troubleshooting

### Problem 1

Due to the large size of the mCherry-pCAG-donor(R2Tg)-GFP(intron) plasmid, the transfection efficiency may be low, resulting in insufficient number of mCherry^+^ cells to be sorted (related to Steps 9–11).

### Potential solution

Prepare two wells or multiple wells of 24-well plates for cell sorting into one tube, or transfect larger amounts of cells in larger surfaces, such as 12-well or 6-well plates.

### Problem 2

The cells sorted into 12-well plates are not growing well (related to Step 12).

### Potential solution

Incubate 12-well plates with PDL in advance or seed the cells into 24-well plates can help cells grow better.

## Resource availability

### Lead contact

Further information and requests for resources and reagents should be directed to and will be fulfilled by the lead contact, Wei Li (liwei@ioz.ac.cn).

### Technical contact

Technical questions on executing this protocol should be directed to and will be answered by the technical contact, Yangcan Chen (chenyangcan@ioz.ac.cn).

### Materials availability

Plasmids used in this study are available in Addgene and have been listed in the key resources table.

### Data and code availability

This study does not generate any new sequencing data and code.

## Acknowledgments

This work was supported by the National Natural Science Foundation of China (32225030 to W.L.), the National Key Research and Development Program (2019YFA0903800, 2019YFA0110800, 2021YFA0805905, and 2021YFA1101600), the CAS Project for Young Scientists in Basic Research (YSBR-012 to W.L.), the Strategic Priority Research Program of the Chinese Academy of Sciences (XDA16030403 to W.L.), the Beijing Natural Science Foundation (Z230011 to W.L.), the international cooperation project of China Manned Space Program, the China Postdoctoral Science Foundation (2023M743477 to Y.C.), and the Postdoctoral Fellowship Program of CPSF (GZB20230751 to Y.C.).

## Author contributions

W.L., Q.Z., and Y.C. conceived the project and designed the experiments; Y.C., S.L., and Y.H. performed the experiments; W.L. and Q.Z. supervised the research; and W.L., Q.Z., Y.C., S.L., and Y.H. wrote the manuscript.

## Declaration of interests

Q.Z. is a member of the *Cell* advisory board.

## Supplemental information

Table S1. DNA sequences of related primers, synthetic genes and plasmids, related to Step 1 to Step 4
